# Rethinking Lesser Mealworm Management: New Evidence from Two Entomopathogenic Nematodes

**DOI:** 10.3390/insects17060578

**Published:** 2026-06-01

**Authors:** Eirini Karanastasi, Lampros Lamprou, Georgia Anna Tzouda, Christos I. Rumbos

**Affiliations:** 1 Plant Protection Laboratory, Department of Agriculture, School of Agricultural Sciences, 30200 Messolonghi, Greece; up1076926@ac.upatras.gr (L.L.); up1081815@ac.upatras.gr (G.A.T.); 2 Department of Agriculture, International Hellenic University, Alexander University Campus, 57400 Thessaloniki, Greece; crumbos@ihu.gr

**Keywords:** *Alphitobius diaperinus*, *Heterorhabditis downesi*, *Steinernema kraussei*, biological control, temperature-dependent infectivity, poultry facility pests

## Abstract

*Alphitobius diaperinus* (Panzer) (Coleoptera: Tenebrionidae) is a common pest in poultry farms, causing significant economic damage, spreading easily and quickly. The species is resilient, adaptable, and capable of thriving in a variety of environments, and both larvae and adults can hide in hard-to-reach places; thus, control becomes highly challenging. Moreover, the species has developed resistance to commonly used insecticides, increasing the need to adopt integrated pest management strategies. Hence, this study examined the mortality dynamics of *A. diaperinus* larvae exposed to the entomopathogenic nematodes (EPNs) *Heterorhabditis downesi* Stock, Griffin & Burnell 2002 (Rhabditida: Heterorhabditidae) and *Steinernema kraussei* (Steiner, 1923) (Rhabditida: Steinernematidae) across a range of temperatures, dose rates, and exposure intervals. The patterns of larval mortality closely followed the classical EPN infection timeline: limited mortality during the first hours, a pronounced increase between day 2 and 4, and high cumulative mortality after day 8. *Steinernema kraussei* produced moderate early mortality, increasing by day 8, across all tested temperatures, reflecting its relatively slower but effective infection progression. In contrast, *H*. *downesi* induced higher early mortality at moderate temperatures and stronger dose-dependent responses. Temperature significantly modulated infection in both species, with reduced performance at 35 °C, particularly for *H. downesi*, although high-dose treatments achieved substantial mortality. The significant temperature × dose × time interactions align with established EPN biology and previous research on both species and, overall, the results confirm that they both possess strong pathogenic potential against *A. diaperinus*, while their performance characteristics support their suitability as biological control agents for poultry facilities.

## Simple Summary

The lesser mealworm, *Alphitobius diaperinus*, is a widespread pest in poultry farms that causes economic losses and is difficult to control because it hides in small spaces and has developed resistance to many insecticides. This study explored whether two naturally occurring beneficial nematodes could be used as an alternative control method. Specifically, we tested how effectively *Heterorhabditis downesi* and *Steinernema kraussei* kill the larvae under different temperatures, doses, and exposure times. Results showed that both species can successfully infect and kill the pest, with mortality increasing over time, especially after several days. *Steinernema kraussei* acted more slowly but consistently, while *H. downesi* worked faster at moderate temperatures and responded more strongly to higher doses. Temperature influenced their performance, with reduced effectiveness at very high temperatures. Overall, both nematodes showed strong potential as biological control agents. These findings support the use of environmentally friendly pest management strategies in poultry production, helping reduce reliance on chemical insecticides and contributing to more sustainable and safer food systems.

## 1. Introduction

The darkling beetle, litter bug or lesser mealworm, *Alphitobius diaperinus* (Panzer) (Coleoptera: Tenebrionidae), is the most common pest in poultry houses, while posing a significant threat in other agricultural, livestock and industrial settings, as it can also infest stored grain commodities and other stored feeds, insulating materials, etc. [1,2]. In natural environments, this species is commonly associated with bird nests, where it lives as a nest commensal and utilizes leftover organic material and debris from hosts including pigeons and sparrows [3,4]. Comparable behavior has also been reported in cave ecosystems, where the beetle develops on detritus and bat guano [5]. *Alphitobius diaperinus* is a highly resilient pest with the ability to spread quickly, developing high populations, thus causing significant economic losses and creating unsanitary conditions if not controlled properly and in a timely manner [6]. On the other hand, when broilers consume adults or the larvae of *A. diaperinus*, they tend to present lower weight gain and feed conversion ratio [7]. According to Guillebeau et al. [8], the control cost of darkling beetle infestations in poultry farms in Georgia (USA) in 2006 was estimated at $4,516,000. Moreover, the species is known as a vector or source for several poultry pathogens that cause significant diseases such as aspergillosis, Marek’s disease, infectious bursal disease (IBD), Newcastle disease and coccidiosis [1]. It may also aid the transmission of food-borne pathogens such as *Salmonella* and *Campylobacter* to broilers [9,10,11] and, occasionally, serves as an intermediate host for nematodes and fowl tapeworms that infest poultry [12].

Consequently, controlling *A. diaperinus* is of primary importance, particularly in poultry production systems, where the species acts as both a structural pest and a vector of economically significant pathogens [6,11]. Its management is challenging because it is hardy, highly adaptable, and capable of thriving across a wide range of environmental conditions [6]. Current control programs typically integrate multiple strategies. A foundational component is sanitation: regular removal of spilled feed, thorough cleaning of litter, proper waste management, and sealing of cracks and crevices are essential because these areas provide harborage and nutrient resources that sustain beetle populations [7,9]. Environmental manipulation is also valuable: *A. diaperinus* exhibits tolerance to moderate thermal fluctuations, but exposure to extreme temperatures, either high (>45 °C) or below the freezing point, can significantly reduce survival, especially during storage or downtime between flocks [6,13]. In addition, pheromone-based trapping has emerged as a useful tool for monitoring population density and, in some cases, contributing directly to population suppression through aggregation lure-based capture systems [14]. Collectively, these integrated approaches provide the foundation for effective, long-term management of *A. diaperinus* in poultry facilities.

Chemical control, primarily through the application of registered insecticides with relatively short residual activity, remains the most used tactic for managing *A. diaperinus* in poultry houses [6]. For this purpose, several carbamate- and organophosphate-based formulations are applied as wettable powders, liquid sprays, or baits targeting surfaces, cracks, walls, ceilings, insulation, and support structures within and around poultry facilities [8]. However, *A. diaperinus* populations have demonstrated a capacity to develop insecticide resistance, a pattern consistent with resistance evolution in other synanthropic pests exposed to repeated chemical treatments [15]. Consequently, rotating active ingredients with different modes of action, as well as combining adulticides with larvicides, is recommended to delay resistance development and enhance overall treatment efficacy [6]. Ultimately, chemical control is most effective when embedded within integrated pest management (IPM) frameworks, in which sanitation, mechanical exclusion, environmental manipulation, and biological agents (including entomopathogenic nematodes and fungi) are strategically combined to achieve sustainable population suppression [16,17,18].

Biological control, i.e., employing natural enemies such as birds, beneficial insects, or entomopathogenic fungi, can help regulate *A. diaperinus* populations. Fungal entomopathogens, such as *Beauveria bassiana* and *Metarhizium anisopliae*, have been found effective against several insect pests, including *A. diaperinus* larvae, infecting them upon contact, leading to death within a few days [19,20]. Bacterial agents like *Bacillus thuringiensis* have also been investigated for their potential to control *A. diaperinus*, although relevant published data remain scarce [21]. Predatory beetles, such as *Carcinops* spp., can prey on *A. diaperinus* larvae and adults and are part of the natural ecosystem in some regions, though their practical application in poultry houses has been minimally studied [22]. Parasitic wasps, particularly those in genera like *Hymenoepimecis*, parasitize beetles and other insects; however, they are rarely used in poultry farms for *A. diaperinus* control and remain largely unexplored in this context [6]. Recently, entomopathogenic nematodes (EPNs) of the genera *Steinernema* and *Heterorhabditis* have received considerable research attention, as they can infect and kill *A. diaperinus* larvae and occasionally adults under laboratory conditions [21,23]. Given that their natural habitat is soil, EPNs could be effectively applied in bedding or manure, where larvae are typically found, becoming promising biocontrol agents [19,21]. To date, biocontrol research with EPN against *A. diaperinus* has focused on the use of the three most common nematode species, i.e., *S. carpocapsae*, *S. feltiae* and *H. bacteriophora* [16,17,18,23,24,25,26], and most results conclude that *Steinernema* species are more efficient, especially against the larvae of the litter bug at temperatures around 25 °C. Nevertheless, since the success of EPNs depends on multiple biological parameters, such as nematode tactics, insect behavior and insect susceptibility to the nematode symbiotic bacterial species or strain, i.e., on the specific nematode–insect relationship, and all these depend concurrently on the prevailing conditions, other possible EPN species should be investigated with respect to their effectiveness. Therefore, evaluating *H. downesi* and *S. kraussei* against *A. diaperinus* addresses a specific knowledge gap by extending EPN screening beyond the most commonly tested species and by assessing two isolates with biological traits potentially suited to cryptic larval habitats.

During the present study, we studied the possible lethal effect of two isolates of *Heterorhabditis downesi* Stock, Griffin & Burnell 2002 (Rhabditida: Heterorhabditidae) and *Steinernema kraussei* (Steiner, 1923) (Rhabditida: Steinernematidae), against single *A. diaperinus* larvae exposed at six EPN doses, for two, four and eight days, and mortality was recorded at 25 °C, 30 °C and 35 °C. The two EPN species were selected based on published data showing their efficacy against other coleopteran species occupying cryptic environments such as *Hylobius abietis* L. (Coleoptera: Curculionidae) [27,28,29] or species that prefer performing in the dark such as *Otiorhynchus* spp. [30,31,32].

*Heterorhabditis downesi*, a cruiser species known as the Irish strain of *H. megidis*, is a newcomer in the market and has been reported to perform better in sandy soils in the transect from the front of the dunes to the grassland, at temperatures between 8 °C and 35 °C. It presents good persistence and a broad host range, which comprises vine and pine weevil, chafer grubs and cutworms [33], while *Steinernema kraussei* is also considered to adopting a “cruise” foraging strategy, which is ideal for locating subterranean sedentary insects [34,35,36].

## 2. Materials and Methods

### 2.1. Alphitobius diaperinus

The *A. diaperinus* larvae for the trials derived from the insect farm of the Plant Protection Laboratory, University of Patras, where a species population is maintained in semi-dark conditions, at ~26 °C and 65% relative humidity (RH). The insect is reared in 34 × 24 × 16 cm plastic containers lined with a mixture of wheat bran (previously placed at −18 °C for 48 h, then at 50 °C for 48 h to eradicate other possibly present arthropods), yeast and soy protein (6:1:1). Subsequently, one halved apple is added in each container to provide moisture and a polystyrene chunk to serve as a protected pupation site [13].

To obtain the larvae for the trials, unsexed adult individuals were transferred to similar containers lined with flour, for 4 days at 26 ± 1 °C, 55% RH for oviposition [37]. Thereafter, the flour was sieved through a 250 μm sieve and the eggs transferred into glass vials filled with the rearing mixture. The vials were dated so that each one contained coeval larvae.

### 2.2. Entomopathogenic Nematode Isolates

*Heterorhabditis downesi* and *S. kraussei* were originally provided by Bio-insecta (Nea Silata, Nea Silata, Greece) and Agrimore S.A. (Kalochori, Thessaloniki, Greece), respectively. Then, the nematodes were multiplied in larvae of the greater wax moth, *Galleria mellonella* (L.) (Lepidoptera: Pyralidae), at 25 °C [38]. Emerging populations were stored at ~10 °C in cell culture flasks until use, but prior to each trial, vitality was examined under a stereomicroscope. Nevertheless, none was used if it had been stored for more than 4 weeks.

### 2.3. Bioassays

The trials were conducted following the protocol of Karanastasi et al. [23]. Single *A. diaperinus* larvae were transferred to separate 30 mm plastic Petri dishes, lined with filter paper. The EPN doses used in the present study were 10, 50, 100, 500, 1000, and 5000 infective juveniles (IJs)/larva, as previously described [16,23,39]. For each dose, EPN species and tested temperature (25, 30, and 35 °C), the bioassays comprised 3 × 30 Petri dishes, each containing one unsexed small larva (<7 mm), at 65% RH. Small larvae were selected to ensure developmental uniformity throughout the 8-day observation period, as preliminary observations indicated that larger larvae frequently entered pupation during the exposure period, which could confound the assessment of larval mortality and EPN infection. Each larva was inoculated with a given EPN dose, applied with 100 μL tap water. The EPN doses were freshly prepared for each replication. Control Petri dishes were inoculated with 100 μL tap water.

Larval mortality was recorded after 2, 4 and 8 d of exposure. To ensure that motionless larvae were diseased, they were poked with a brush under a stereomicroscope for status confirmation. Dead individuals were transferred to white traps, distinctly for each bioassay. EPN infection was confirmed with the emergence of EPN IJs within the following two weeks.

Additionally, if any larvae turned into pupae before the bioassay completion, i.e., before the 8 d lapse, these were singly maintained for observation at the respective temperature regime.

The whole experiment employed 3510 individual larvae, i.e., 30 larvae × (2 EPN species + 1 control) × 6 EPN doses × 3 repetitions × 3 temperatures.

### 2.4. Statistical Analysis

Mortality values were corrected using Abbott’s formula [40]. Before analysis, the dataset was examined for normality with the Shapiro–Wilk test and for homogeneity of variance with Levene’s test. Because mortality was recorded from the same vials after 2, 4, and 8 d of exposure, the data were evaluated with a repeated-measures ANOVA, treating exposure time as the repeated factor and nematode formulation, dose rate, and temperature as fixed effects. The assumptions underlying the repeated-measures ANOVA, including sphericity, were evaluated and met. All analyses were performed using JMP 7 (SAS Institute Inc., Cary, NC, USA). For each nematode isolate and exposure period, and within each dose level, an ANOVA was conducted to assess temperature effects. Likewise, for each exposure time and formulation, and within each temperature, ANOVA was used to test for differences among dose rates. Post hoc comparisons were made using Tukey’s HSD test with a significance threshold of 5%.

## 3. Results

In all cases, control mortality did not exceed 20%. Control larvae originated from healthy, coeval laboratory cohorts and were inspected before use; no visibly injured, weakened, or abnormally behaving larvae were included in the assays. Therefore, the observed control mortality was not attributed to poor initial larval condition, but was more likely associated with the relatively long 8-day observation period and the stress imposed by individual handling and confinement under bioassay conditions. For this reason, mortality data were corrected using Abbott’s formula before statistical analysis. *Alphitobius diaperinus* larval mortality was significantly affected by the exposure interval (F = 656.3, *p* < 0.001) and most main effects, i.e., nematode isolate (F = 43.9, *p* < 0.001) and dose rate (F = 7.1, *p* < 0.001), with the exception of temperature (F = 1.4, *p* = 0.242), whereas all associated interactions were also significant (Table 1).

### 3.1. Steinernema kraussei

Overall, *S. kraussei* showed a clear time-dependent increase in mortality across temperatures, although the dose response was not strictly linear under all conditions.

**Figure 1:**
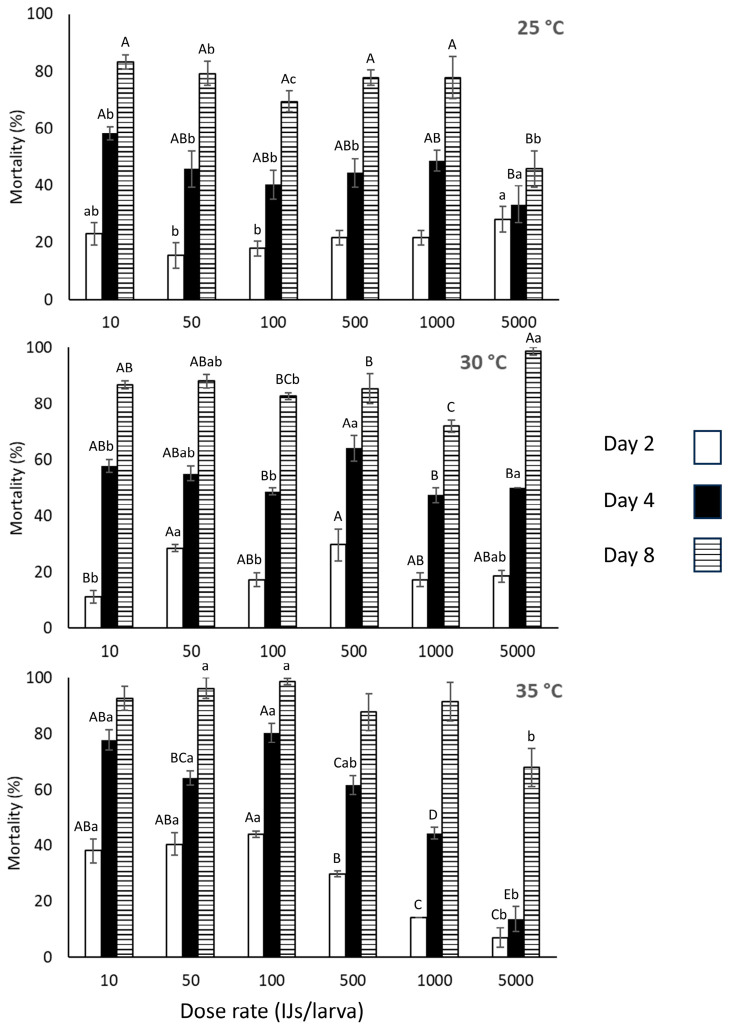
Mean mortality ± SE of *Alphitobius diaperinus* larvae exposed for 2, 4 and 8 days to a *Steinernema kraussei* isolate applied at six dose rates (10, 50, 100, 500, 1000 and 5000 IJs/larva) at three temperatures (25, 30 and 35 °C). For each exposure interval, within each temperature, means followed by the same uppercase letter do not differ significantly (in all cases df = 5, 17; Tukey HSD test at *p* = 0.05). For each exposure interval, within each dose rate, means followed by the same lowercase letter are not significantly different (in all cases, df = 2, 8; Tukey HSD test at *p* = 0.05). Where no letters exist, no significant differences were noted.

After 2 d of exposure at 25 °C, mortality levels of *A. diaperinus* larvae did not exceed 28.2% for all treatments (Figure 1). Mortality rates increased with time reaching 58.3 and 83.3% after 4 and 8 d of exposure, respectively. A similar pattern was observed for mortality rates at 30 °C, where the highest mortality rate at the shortest exposure interval (2 d) was achieved after the application of 500 IJs/larva (29.6%). After 4 d of exposure, larval mortality further increased and ranged between 47.4 and 64.1%. At the final evaluation point (8 d), more than 72% of the larvae died in all treatments, with the highest mortality level (98.7%) being recorded for the highest dose (5000 IJs/larva). When *S. kraussei* was applied at 35 °C, mortality levels after 2 d of exposure fluctuated between 7.1 and 44.1% and were further increased after 4 d of exposure, ranging between 13.6 and 80.2%. Mortality was higher than 67.9% for all treatments at the final evaluation point (8 d), with the highest mortality level being recorded after the application of 100 IJs/larva (98.7%).

It should be noted that mortality responses in *S. kraussei* were not strictly dose-dependent under all temperature regimes. In particular, at 35 °C, the highest mortality values were recorded at lower or intermediate dose rates rather than at the highest dose. This non-monotonic pattern suggests that, under thermal stress, increasing IJ concentration does not necessarily result in proportionally higher mortality and may reflect density-dependent effects, altered IJ activity, or reduced infection efficiency at excessive concentrations.

### 3.2. Heterorhabditis downesi

Overall, *H. downesi* produced faster early mortality than *S. kraussei*, particularly at moderate temperatures and higher dose rates.

**Figure 2:**
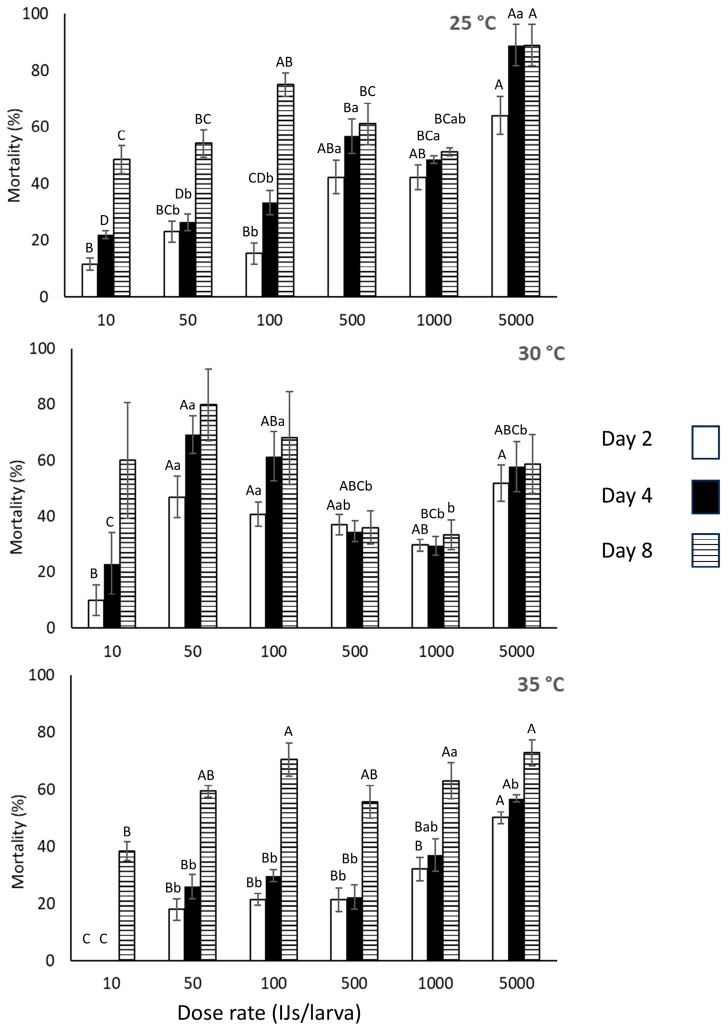
Mean mortality ± SE of *Alphitobius diaperinus* larvae exposed for 2, 4 and 8 days to a *Heterorhabditis downesi* isolate applied at six dose rates (10, 50, 100, 500, 1000 and 5000 IJs/larva) at three temperatures (25, 30 and 35 °C). For each exposure interval, within each temperature, means followed by the same uppercase letter do not differ significantly (in all cases df = 5, 17; Tukey HSD test at *p* = 0.05). For each exposure interval, within each dose rate, means followed by the same lowercase letter are not significantly different (in all cases, df = 2, 8; Tukey HSD test at *p* = 0.05). Where no letters exist, no significant differences were noted.

When *H. downesi* was applied at 25 °C, mortality levels after 2 d of exposure ranged between 11.4 and 64.1%, increasing progressively with higher dose rates (Figure 2). After 4 d of exposure at the same temperature, mortality levels followed the same pattern and ranged between 22.2 and 88.9%, the application of the higher dose rates resulting in significantly higher mortality compared to the lower doses. The number of dead *A. diaperinus* larvae slightly further increased at the longer exposure interval (8 d); however, complete control (100% mortality) was not achieved for any treatment. At 30 °C, the mortality rate 2 d after the application of 10 IJs/larva (9.9%) was significantly lower than the respective figures for most treatments, i.e., 50 (46.9%), 100 (40.7%), 500 (37.0%) and 5000 IJs/larva (51.8%). After 4 d of exposure, mortality rates between 23.1 and 69.2% were recorded and were further increased after 8 d, reaching 80% mortality for the 50 IJs/larva treatment. Finally, after application of *H. downesi* at 35 °C, no mortality (0%) was recorded at the lowest dose rate (10 IJs/larva) after 2 and 4 d of exposure. However, at the first evaluation interval, mortality gradually increased with the increase in the dose rate and reached 17.9, 21.4, 21.4, 32.1 and 50% after the application of 50, 100, 500, 1000 and 5000 IJs/larva, respectively. After 4 d of exposure, 56.8% of *A. diaperinus* larvae were dead at the highest dose rate (5000 IJs/larva), significantly more than the percentage of dead larvae at the rest of the treatments. A similar trend was observed after 8 d of exposure, with mortality rates reaching 72.8% at the highest dose rate (5000 IJs/larva).

## 4. Discussion

The present study demonstrates that the mortality of *A. diaperinus* larvae exposed to *S. kraussei* and *H. downesi* is governed by strong interactive effects among temperature, dose, and exposure time. These interactions are statistically robust, as evidenced by highly significant three-way interactions in the repeated-measures ANOVA. Such interactions indicate that no single factor alone determines EPN efficacy; instead, infection success emerges from the combined influence of environmental conditions, propagule density, and the progression of the nematode–bacterium lifecycle. This framework aligns with established models of EPN pathogenicity, in which IJs locate and penetrate hosts rapidly, but lethal septicemia requires 48–72 h to develop [41]. Consequently, low initial mortality followed by pronounced increases with time represents a classical infection trajectory, a pattern consistently reproduced in our datasets for both nematode species.

### 4.1. Temporal Patterns and Infection Chronology

Across all temperatures and doses, mortality at 2 d remained low for both species. For *S. kraussei*, early mortality at 25–30 °C remained generally below 30%, even at higher doses, while *H. downesi* showed slightly elevated early mortality at moderate and high doses but still reflected incomplete infection at this early stage. These values correspond to the developmental period in which IJs have penetrated, but the symbiotic bacteria (*Xenorhabdus* in *Steinernema*, *Photorhabdus* in *Heterorhabditis*) have not yet reached systemic densities capable of inducing full septicemia. Previous studies have similarly reported this lag phase, with Fraser et al. [42] and Downes and Griffin [43] observing minimal host mortality within the first 24–36 h even under optimal conditions.

By 4 d, mortality in our experiment increased sharply for both nematode species, particularly at moderate and high doses. This increase corresponds with the well-known septicemic phase of infection, during which bacterial proliferation and toxin production rapidly compromise host physiological systems. The strong dose-dependence observed in our study is entirely consistent with the findings of Koppenhöfer and Kaya [44], who showed that higher IJ densities accelerate the host encounter rate and trigger earlier bacterial release, thus shortening the time to death. The near-complete mortality reached by 8 d in most treatments also matches the expected completion of the infection cycle, during which first-generation adults develop and new IJs begin to emerge [45]. These temporal dynamics confirm that the mortality patterns observed here reflect the classical physiological sequence of EPN infection.

### 4.2. Species-Specific Performance: Comparing S. kraussei and H. downesi

Despite the broadly similar infection timelines, differences between species were pronounced and matched theoretical expectations and prior studies. *Heterorhabditis downesi* consistently produced higher early mortality and stronger dose responses at optimal temperatures (25–30 °C). This is characteristic for heterorhabditids, which typically kill hosts more rapidly due to the aggressive virulence of *Photorhabdus* bacteria [43,46]. Early mortality observed at higher doses, reaching up to 40–60% in some treatments by 2 d, aligns with the rapid killing times frequently reported for this species [47,48].

The faster mortality caused by *H. downesi* at moderate temperatures may also reflect differences in the associated bacterial symbionts. Following host penetration, heterorhabditids release *Photorhabdus* bacteria, which proliferate rapidly, produce insecticidal toxins and immune-suppressive factors, and induce septicemia. In contrast, *S. kraussei* is associated with *Xenorhabdus*, and its slower early mortality may reflect differences in bacterial proliferation, toxin expression, or host immune suppression. Because bacterial dynamics and host immune responses were not directly measured in the present study, this interpretation should be considered mechanistic but inferential.

*Steinernema kraussei* exhibited slower early killing but high cumulative mortality. Even at elevated temperatures, where its early performance was muted, mortality at 8 d often exceeded 80–95% at moderate and high doses. These findings match previous characterizations of *S. kraussei* as a species with robust infectivity but comparatively slower septicemic progression [35,36]. The strong late-stage mortality in our experiment underscores its ability to achieve substantial control when exposure times are sufficiently long, consistent with the view that EPN behavioral traits from dispersal to host exploitation can strongly influence fitness and biocontrol efficacy [49].

### 4.3. Thermal Effects on Infection Success

Temperature had predictable and biologically interpretable effects on nematode performance. For both EPN species, mortality at 25 °C and 30 °C followed the expected infection trajectory with strong cumulative mortality. However, performance at 35 °C was consistently reduced, particularly for *H. downesi*, which showed slowed infection, diminished early mortality, and incomplete control at the end of the observation period. This pattern is strongly aligned with earlier findings demonstrating that temperatures approaching the upper thermal threshold of heterorhabditids impair IJ mobility, reduce host penetration, and suppress *Photorhabdus* growth [47,50].

For *S. kraussei*, although early mortality at 35 °C was variable and often low, mortality by day 8 remained high at moderate and high doses. This delayed but cumulative mortality reflects the thermal profile reported in the literature, where *S. kraussei* maintains infectivity across a broad temperature range but exhibits reduced early efficacy at high temperatures [51]. The species’ known specialization for cooler climates, including successful activity below 10 °C, makes its relative resilience at 35 °C noteworthy but not unexpected.

The reduced performance observed at 35 °C also has direct practical implications for poultry-house applications, particularly in warmer climates or during hot periods. Based on the present findings, these isolates may be better suited for application during cooler periods, between production cycles, or in poultry facilities where temperature and moisture can be partially controlled. Under warmer conditions, application timing, substrate moisture, and dose optimization are likely to be critical for maintaining EPN efficacy.

### 4.4. Dose-Dependent Responses

Dose was a significant factor at all time points and temperatures. Higher doses led not only to higher overall mortality but also to faster mortality expression, particularly for *H. downesi*. This is consistent with numerous studies demonstrating that higher IJ densities increase the probability of host encounter and accelerate septicemia development [44,45]. The significant dose × time interactions in our ANOVA underscore the dynamic nature of dose effects: early infection is highly dose-sensitive, while late-stage mortality saturates, reflecting the finite number of susceptible hosts.

Although dose was a significant factor overall, the response was not uniformly linear across all treatments. This was especially evident for *S. kraussei* at 35 °C, where lower or intermediate IJ concentrations produced higher mortality than the highest dose. Such non-monotonic responses have biological plausibility in EPN systems, where high IJ densities may increase competition, interfere with host-seeking behavior, or reduce infection efficiency under stressful thermal conditions. Therefore, the present findings indicate that optimal efficacy may depend not only on applying higher numbers of IJs, but also on matching dose rate with temperature, host stage, and exposure duration.

### 4.5. Integration with Prior EPN Research

The patterns observed here are broadly consistent with previous studies on both species. *Heterorhabditis downesi* has been shown to perform strongly at moderate temperatures but to suffer under thermal extremes [47], while *S. kraussei* exhibits slower killing but high eventual virulence [36]. The reduced performance at 35 °C for both species reflects known constraints on IJ survival, bacterial symbiont growth, and the stability of the nematode–bacterium mutualism under thermal stress [50,51]. Collectively, our results reinforce the biological principles identified in the classical EPN literature and extend them to a novel, economically important host species.

### 4.6. Implications for Biological Control of A. diaperinus

Taken together, the findings of this study suggest that both *S. kraussei* and *H. downesi* have strong potential as biological control agents against *A. diaperinus*, provided that environmental conditions and application rates are optimized. In poultry houses, temperatures typically range from 25 to 30 °C, conditions under which both nematodes performed well in our experiment. *Heterorhabditis downesi* offers faster action and higher early mortality, which may be advantageous in acute infestation scenarios, while *S. kraussei* provides reliable long-term control even when early infection rates are moderate. The strong dose responses observed here indicate that achieving adequate IJ densities will be essential for consistent performance, particularly at marginal temperatures. These results collectively highlight the value of integrating EPN-based strategies into the management programs for lesser mealworm infestations, particularly when chemical control options are limited or resistance is emerging.

However, the translation of these results to poultry-house conditions should be considered with caution. It should also be noted that the present bioassays were conducted on filter paper under standardized laboratory conditions. This approach is appropriate for assessing basic pathogenicity, but it does not reproduce the physical and chemical complexity of poultry litter or manure, where *A. diaperinus* larvae normally occur. Poultry litter represents a heterogeneous three-dimensional substrate with organic matter, variable moisture, microbial activity, and possible ammonia effects, all of which may influence IJ survival, movement, and host location. Therefore, the performance of these cruiser-type nematodes may differ under litter conditions compared with filter paper. Future studies should evaluate these isolates in sterilized and non-sterilized poultry litter to bridge the gap between laboratory pathogenicity and practical field efficacy.

The present study focused exclusively on larvae; therefore, no conclusions can be drawn regarding adult susceptibility. This represents an important area for future investigation, as adult *A. diaperinus* individuals also contribute to pathogen transmission, population persistence, and structural damage in poultry facilities. Future studies should therefore evaluate the efficacy of these isolates against adult beetles under both standardized laboratory conditions and more realistic litter-based bioassays.

## 5. Conclusions

This study demonstrates that *S. kraussei* and *H. downesi* can induce substantial mortality in *A. diaperinus* larvae, with infection success shaped by strong interactions among temperature, dose, and exposure time. Both species followed the classical EPN infection trajectory, characterized by low early mortality, steep increases between 2 and 4 d, and high cumulative mortality by 8 d, confirming that the observed patterns reflect core biological processes governing EPN pathogenicity. *Heterorhabditis downesi* exhibited faster and more aggressive early killing, particularly at moderate temperatures and higher doses, whereas *S. kraussei* produced slower initial effects but high late-stage mortality, even under mildly stressful thermal conditions. Temperature effects aligned with the species’ known thermal biology, with optimal performance at 25–30 °C and reduced infectivity at 35 °C. Dose-dependent increases in mortality further underscored the importance of adequate IJ densities for efficient host suppression.

Overall, these results indicate that both EPN species possess strong potential as biological control agents against *A. diaperinus* larvae under temperature regimes typical of poultry production systems. *Heterorhabditis downesi* may be particularly suited for rapid suppression of high-density larval populations, whereas *S. kraussei* provides consistent long-term efficacy across a broader range of environmental conditions. Integrating these EPNs into IPM programs could reduce reliance on chemical insecticides and mitigate resistance development, offering an environmentally sustainable and biologically robust option for managing lesser mealworm infestations.

## Figures and Tables

**Table 1:** Repeated-measures ANOVA parameters for main effects and associated interactions for mortality levels of *Alphitobius diaperinus* larvae exposed for 2, 4 and 8 days to two entomopathogenic nematode isolates (*Steinernema kraussei* and *Heterorhabditis downesi*) applied at six dose rates (10, 50, 100, 500, 1000 and 5000 IJs/larva) at three temperatures (25, 30 and 35 °C) (error df: 72).

Source	df	F	*p*
*Source between variables*			
All between	35	12.5	<0.001
Intercept	1	4998.3	<0.001
Nematode isolate	1	43.9	<0.001
Dose rate	5	7.1	<0.001
Temperature	2	1.4	0.242
Nematode isolate × Dose rate	5	33.8	<0.001
Nematode isolate × Temperature	2	22.4	<0.001
Dose rate × Temperature	10	5.9	<0.001
Nematode isolate × Dose rate × Temperature	10	8.3	<0.001
*Source within variables*			
Within interactions	70	8.4 *	<0.001
Exposure	2	656.3	<0.001
Exposure × Nematode isolate	2	113.9	<0.001
Exposure × Dose rate	10	3.9 *	<0.001
Exposure × Temperature	4	9.0 *	<0.001
Exposure × Nematode isolate × Dose rate	10	11.1 *	<0.001
Exposure × Nematode isolate × Temperature	4	10.0 *	<0.001
Exposure × Dose rate × Temperature	20	2.7 *	<0.001
Exposure × Nematode isolate × Dose rate × Temperature	20	4.1 *	<0.001

* Wilks’ Lambda.

## Data Availability

The original contributions presented in this study are included in the article. Further inquiries can be directed to the corresponding author.
